# Cigarette smoke extract counteracts atheroprotective effects of high laminar flow on endothelial function

**DOI:** 10.1016/j.redox.2017.04.008

**Published:** 2017-04-07

**Authors:** Sindy Giebe, Natalia Cockcroft, Katherine Hewitt, Melanie Brux, Anja Hofmann, Henning Morawietz, Coy Brunssen

**Affiliations:** a Division of Vascular Endothelium and Microcirculation, Department of Medicine III, Medical Faculty Carl Gustav Carus and University Hospital Carl Gustav Carus Dresden, Technische Universität Dresden, Dresden, Germany; b Research & Development, British American Tobacco, Southampton, United Kingdom

**Keywords:** Cigarette smoke extract, Endothelial dysfunction, Endothelial cells, Shear stress and laminar flow, NRF2, Monocyte adhesion

## Abstract

Tobacco smoking and hemodynamic forces are key stimuli in the development of endothelial dysfunction and atherosclerosis. High laminar flow has an atheroprotective effect on the endothelium and leads to a reduced response of endothelial cells to cardiovascular risk factors compared to regions with disturbed or low laminar flow. We hypothesize that the atheroprotective effect of high laminar flow could delay the development of endothelial dysfunction caused by cigarette smoking. Primary human endothelial cells were stimulated with increasing dosages of aqueous cigarette smoke extract (CSEaq). CSEaq reduced cell viability in a dose-dependent manner. The main mediator of cellular adaption to oxidative stress, nuclear factor erythroid 2-related factor 2 (NRF2) and its target genes heme oxygenase (decycling) 1 (HMOX1) or NAD(P)H quinone dehydrogenase 1 (NQO1) were strongly increased by CSEaq in a dose-dependent manner. High laminar flow induced elongation of endothelial cells in the direction of flow, activated the AKT/eNOS pathway, increased eNOS expression, phosphorylation and NO release. These increases were inhibited by CSEaq. Pro-inflammatory adhesion molecules intercellular adhesion molecule-1 (ICAM1), vascular cell adhesion molecule-1 (VCAM1), selectin E (SELE) and chemokine (C-C motif) ligand 2 (CCL2/MCP-1) were increased by CSEaq. Low laminar flow induced VCAM1 and SELE compared to high laminar flow. High laminar flow improved endothelial wound healing. This protective effect was inhibited by CSEaq in a dose-dependent manner through the AKT/eNOS pathway. Low as well as high laminar flow decreased adhesion of monocytes to endothelial cells. Whereas, monocyte adhesion was increased by CSEaq under low laminar flow, this was not evident under high laminar flow.

This study shows the activation of major atherosclerotic key parameters by CSEaq. Within this process, high laminar flow is likely to reduce the harmful effects of CSEaq to a certain degree. The identified molecular mechanisms might be useful for development of alternative therapy concepts.

## Highlights

• CSEaq induces anti-oxidative enzymes and adhesion molecules in endothelial cells.
• CSEaq increases monocyte adhesion to endothelial cells under low laminar flow.
• Protective effects of high laminar flow on wound healing are inhibited by CSEaq.
• Protective activation of PI3K/AKT/eNOS pathway by high laminar flow is inhibited by CSEaq.

## 1 Introduction

Tobacco smoking is one of the most important risk factors of atherosclerosis, as the underlying process of heart attack and stroke [1]. Endothelial cells play a critical role in the control of vascular function. Lack of regulatory capacity leads to dysregulation of vascular homeostasis, inflammation, diminished vascular wall remodeling and development of endothelial dysfunction. Endothelial dysfunction is an early event in atherosclerosis and has prognostic value for future cardiovascular events. It is characterized by an imbalance between vasodilation and vasoconstriction, a pro-inflammatory phenotype of the endothelial cells, increased adhesion of monocytes and reduced bioavailability of nitric oxide (NO) [2].

Stimulation with CSEaq leads to development of endothelial dysfunction and induces changes in gene expression of several genes, including the key enzyme endothelial nitric oxide synthase 3 (eNOS) [3], [4], [5], [6], [7], [8]. Low or disturbed blood flow has a pro-atherogenic effect on the endothelium at medium and large arteries, promoting differentiation of endothelial and smooth muscle cells into pro-inflammatory phenotypes [9], [10], [11], [12], [13]. This type of flow typically occurs on the near side of arterial vessel branching [14]. Referring to the "Response to Injury" hypothesis, lesions of atherosclerosis arise as a result of some form of "injury" to arterial endothelium [15]. Generation of a plaque within the vessel wall further disturbs the local flow profile [16]. High laminar flow has an atheroprotective effect on the endothelium, activating signal transduction as well as gene and protein expressions that play important roles in vascular homeostasis. In response to laminar flow, the release of vasoactive substances such as nitric oxide (NO), decreases permeability to plasma lipoproteins as well as the adhesion of leukocytes [17]. These molecular changes in response to laminar flow reduce the susceptibility of endothelial cells to cardiovascular risk factors compared to disturbed flow conditions [18]. Differences in flow are immediately recognized by endothelial cells, in that mechanical forces are transduced into biochemical signals [19]. This leads to changes in gene expression of different cellular systems [20].

The nuclear factor, erythroid 2 like 2 (NRF2) system is known to be activated by laminar flow and by oxidative stress. NRF2 is the main mediator of cellular adaptation to oxidative stress and can be directly regulated by cigarette smoke in vascular and blood cells [21], [22]. Upon activation, NRF2 binds to AREs (Antioxidant Response Elements) in the regulatory regions of detoxifying and antioxidant genes, such as heme oxygenase (decycling) 1 (HMOX1) or NAD(P)H quinone dehydrogenase 1 (NQO1) and modulates their transcription [23], [24]. Further important molecular players in this context are eNOS and adhesion molecules [12], [13], [14], [25]. The expression of eNOS can be modulated by laminar flow [26]. Moreover, the AKT pathway activates eNOS through direct phosphorylation of AKT at Ser473 in response to laminar flow [27], [28], [29]. Adhesion molecules mediate the adhesion of monocytes to endothelial cells; a key mechanism in the pathogenesis of atherosclerosis [30], [31]. However, the molecular mechanisms relating to how cigarette smoke extract (CSEaq) and different types of flow regulate monocyte adhesion to endothelial cells and wound healing as a model of vascular wall remodeling are not well understood.

We hypothesize that the atheroprotective effect of high laminar flow could delay the development of endothelial dysfunction caused by detrimental effects of cigarette smoking. Therefore, we analyzed key parameters of endothelial function, monocyte adhesion and wound healing in response to CSEaq under different laminar flow conditions. Identification of the underlying molecular mechanisms might be useful as for development of future therapy concepts.

## 2 Materials and methods

### 2.1 Cell culture

The collection of primary human umbilical vein endothelial cells (HUVEC) was approved by the ethical review board of the Medical Faculty Carl Gustav Carus of the TU Dresden (EK124082003). Primary cultures of HUVEC were isolated using 0.5% collagenase II solution (Worthington Biochemical Corp., Lakewood NJ, USA) [32], [33], [34]. Isolated HUVEC were cultured on 2% gelatin-coated plates in Medium 199 (Thermo Fisher Scientific, Waltham, MA, USA), supplemented with 10% fetal calf serum (Biochrom, Berlin, Germany), 0.5% self-isolated retina calf eye growth supplement [35], 100,000 U/l penicillin (Thermo Fisher Scientific, Waltham, MA, USA), 100 mg/l streptomycin (Thermo Fisher Scientific, Waltham, MA, USA) and 250 mg/l Fungizone (Thermo Fisher Scientific, Waltham, MA, USA). HUVEC were freshly isolated on a weekly manner and only used for experiments up to passage 2. Monocytic cell line THP-1 (ATCC# TIB-202) was provided by the Department of Cardiology, TU Dresden. Cultivation occurred in RPMI-1640 (Thermo Fisher Scientific, Waltham, MA, USA) containing 10% fetal calf serum. Primary human monocytes were isolated from buffy coats by a two-step density gradient centrifugation and cultured as described previously [36]. Human microvascular endothelial cells (HMEC-1, ATCC# CRL-3243) were cultured in Endothelial Cell Growth Medium MV2 (PromoCell, Heidelberg, Germany). Cultivation of all cell types and all experiments were performed in a humidified environment with 5% CO_2_ at 37 °C. All experiments, unless otherwise indicated, were conducted 24 h after reaching confluence (typical cobblestone morphology).

### 2.2 CSEaq production and stimulation

3R4F reference cigarettes were conditioned at 22 °C and 60% relative humidity for 48 h and smoked on a RM20H smoking machine (Borgwaldt-KC, Hamburg, Germany) under ISO standard puffing conditions (1 puff/min; 2 s/puff>35/2/60, vents open; ISO 3308:2012) with the exception of only 8 puffs taken per cigarette instead of smoking the cigarette to a specified butt length. CSEaq was generated by bubbling the smoke from a single cigarette through a glass impinger containing 20 ml of Phenol Red-free M199 medium (without supplements; Thermo Fisher Scientific, Waltham, MA, USA). Prior to treatment with CSEaq, HUVEC were cultivated in Phenol Red-free Medium 199, supplemented with 10% fetal calf serum and 0.5% self-isolated retina calf eye growth supplement for at least 90 min. Stimulation with CSEaq was performed in a concentration range from 10% to 88.3% for up to 48 h with or without application of different types of flow. Each sample was accompanied by a control from the same cell preparation, incubated for the same period of time, without stimulation of CSEaq or application of flow (time-matched controls).

### 2.3 Application of different types of flow

HUVEC were subjected to flow using either a cone-and-plate viscometer [32], [37], [38], [39] or the ibidi pump system (ibidi, Martinsried, Germany) [40]. High laminar flow was defined as 30 dyn/cm^2^ and low laminar flow as 1 dyn/cm^2^. 90 min prior to the application of flow, cells were cultivated in Phenol Red-free Medium 199, supplemented with 10% fetal calf serum and 0.5% growth supplement. In experiments using the cone-and-plate viscometer, 5% dextran was added to increase the viscosity of the medium. Each sample was accompanied by a control from the same cell preparation, incubated for the same period of time, without application of flow (static time-matched controls).

### 2.4 Analysis of cell viability of endothelial cells

The amount of ATP is an indicator of metabolically active cells and directly proportional to the number of cells present in culture [41]. By quantification of the amount of ATP present using CellTiter-Glo® Luminescent Cell Viability Assay (Promega, Mannheim, Germany), the number of viable cells in culture can be determined. HUVEC were stimulated with CSEaq in a concentration range from 10% to 88.3% for up to 48 h under static or flow conditions. Afterwards, the cell viability assay was performed and the emitted luminescence signal was measured using a luminometer. We performed a nonlinear regression analysis of the dose-response curves to calculate the EC_50_ values.

### 2.5 Detection of nitric oxide

Nitric oxide (NO) was measured by Griess reaction as described previously [42]. In brief, 100 µl of supernatant from treated HUVEC was transferred to a 96-well plate. 50 µl of 2% aminobenzenesulphoamide in 2.5% phosphoric acid was added and incubated for 5 min protected from light. Afterwards, 0.2% NED-reagent (N-(1-naphthyl) ethylenediamine dihydrochloride) was added to each well and incubated for 10 min, protected from light. Nitrite release was characterized by an increase in absorbance at 540 nm and compared with a nitrite standard (0–100 µM) using linear regression analysis.

### 2.6 Real-time PCR

Total RNA from treated HUVEC was isolated using High Pure RNA Isolation Kit (Roche Diagnostics, Mannheim, Germany). RNA integrity has been analyzed by Agilent 2100 bioanalyzer using Eukaryote Total Nano 2.6 assay. Reverse transcription of mRNA into cDNA was performed with SuperScript II Reverse Transcriptase according to manufacturer's instructions (Thermo Fisher Scientific, Waltham, MA, USA) using 500 ng of total RNA and random hexamer primers. Quantification was performed by real-time PCR (7500 Fast Real-Time PCR System, Thermo Fisher Scientific, Waltham, MA, USA) using GoTaq qPCR Master Mix (Promega, Mannheim, Germany) with specific primers (Sigma-Aldrich, Munich, Germany/for primer sequences see Table 1). POLR2A was used as reference gene for cDNA content normalization. Amplification started with an initial denaturation step at 95 °C for 10 min, followed by 40 cycles of denaturation at 95 °C for 15 s, specific annealing and extension for each gene for 60 s. Melt‐curve analysis was performed following every run to ensure a single amplified product in each reaction. Analysis of the raw data was performed with the 7500 Software Version 2.06 (Applied Biosystems by Life Technologies, Darmstadt, Germany). Data were evaluated using a mathematical model of relative expression ratio in real-time PCR under constant reference gene expression [43].

### 2.7 Analysis of NQO1 antioxidant response element (ARE)

Analysis of NQO1 antioxidant response element (ARE) was performed as described previously. NQO1-ARE and mutated NQO1-ARE containing pGL4 luciferase reporter vectors were kindly provided by Anna–Liisa Levonen and Hanna Leinonen (University of Eastern Finland, Kuopio, Finland) [34], [44]. In brief, human microvascular (HMEC-1) endothelial cells were transfected using Fugene HD in 24 well plates (Fugene HD: DNA-Ratio =3:1). After 24 h, basal activity was determined by Dual-Luciferase Reporter Assay, or cells were stimulated with CSEaq for additional 24 h. Data were normalized to the activity of a promoter-less firefly luciferase vector pGL4.10[luc2] for determination of basal activity, or to corresponding controls for determination of effects of CSEaq stimulation. In total, 0.5 µg plasmid DNA was used per well. 0.2 µg pGL4.74[hRluc/TK] DNA was used as transfection control per well. All assay reagents were purchased from Promega, Mannheim, Germany.

### 2.8 Western blot

Whole cell extracts were prepared using RIPA Buffer (Cell Signaling, Leiden, Netherlands) including Protease Inhibitor Cocktail (Sigma-Aldrich, Munich, Germany). Prior to short-term phosphorylation studies, cells were rested in basal media without supplements for 60 min. Before lysis, cells were washed twice with ice-cold PBS containing phosphatase inhibitors (Active Motif, Carlsbad, CA, USA). Protein concentration was determined by BCA Protein Assay Reagent (Perbio Science, Bonn, Germany/Thermo Fisher Scientific, Waltham, MA, USA) to ensure equal amounts of protein used for subsequent analysis. Proteins were denaturated for 10 min at 95 °C, separated by 8% SDS-PAGE and transferred to nitrocellulose membranes. Membranes were incubated with the following antibodies: HMOX1 and eNOS (BD Transduction Laboratories, Heidelberg, Germany); NRF2 (Abcam, Cambridge, UK); NQO1, ACTB as a loading control, P-eNOS (Ser1177), AKT and P-AKT (Ser473) (Cell Signaling, Leiden, The Netherlands) for 1 h. Afterwards, membranes were incubated for 1 h with anti-rabbit (Acris, Herford, Germany) or anti-mouse (Life Technologies, Darmstadt, Germany) IgG HRP-conjugated secondary antibodies. Protein expression was detected with Western Lightning Chemiluminescence Reagent Plus (PerkinElmer, Rodgau, Germany) and quantified using AIDA Image Analyzer software (Raytest, Berlin, Germany).

### 2.9 Monocyte-endothelial cell adhesion assay under flow

HUVEC were seeded in a μ-Slide I 0.4 Luer (ibidi, Martinsried, Germany) and allowed to adhere for 4 h under static conditions. After adherence, HUVEC were perfused with Phenol Red-free Medium 199, supplemented with 10% fetal calf serum and 0.5% growth supplement with a flow rate of 30 dyn/cm^2^ until reaching confluence (24 h), followed by 24 h pre-incubation with the indicated type of flow. Subsequently, cells were stimulated for 24 h with CSEaq in combination with or without flow. Human monocytes were cultured in advance in RPMI-1640, supplemented with 10% fetal calf serum. Directly prior to the adhesion assay, monocytes were stained with a 10 µM Cell Tracker Orange CMRA-PBS solution according to the manufacturer's instructions (Life Technologies, Darmstadt, Germany). Fluorescently labeled monocytes were re-suspended in Phenol Red-free Medium 199, supplemented with 0.5% growth supplement. 175,000 monocytes/ml were added to the pre-stimulated endothelial cells. After an incubation period of 20 min, non-adherent monocytes were removed by gentle washing with PBS (Thermo Fisher Scientific, Waltham, MA, USA). Cells were fixed using 4% formaldehyde/PBS solution (Merck, Darmstadt, Germany) for 20 min. Monocyte-endothelial interactions were visualized by 10×10 mosaic fluorescence/phase contrast images, taken using a Zeiss Axio Observer.Z1 ApoTome microscope (Zeiss, Göttingen, Germany). The assay was performed under static or under flow conditions in combination with CSEaq. For evaluation of the experiments, each sample was accompanied by a control from the same cell preparation, incubated for the same period of time, without stimulation of CSEaq or application of flow (static time-matched controls).

### 2.10 Wound healing assay

A wound in the endothelial cell layer is considered as an initial stimulus for the development of atherosclerotic plaques (response-to-injury model) [45]. To mimic this situation in an *in vitro* setup, HUVEC were cultivated in cell culture dishes containing cell culture inserts (ibidi, Martinsried, Germany), to create a defined wound area. Subsequently, cells were exposed to CSEaq under different flow conditions (plate-and-cone viscometer). All experiments were performed in a humidified environment with 5% CO_2_ at 37 °C. Direction of flow was perpendicular to the wound in the cell layer. Each sample was accompanied by a static control without further treatment (static time-matched controls). The increasing closure of the wound was documented for a time period of 10 h and quantified using the Fiji image processing package [46].

### 2.11 Evaluation of data and statistical analysis

Time-matched controls were used for this study. For a series of experiments, basal controls were needed in addition. Data are shown as mean (x-fold or % of time-matched controls or basal controls). If not otherwise stated, time-matched controls were used for normalization of the data. If basal controls were used, time-matched controls are shown as x-fold or % of basal controls in addition. Basal controls represented the status of isolated HUVEC cultures prior to the start of the experiments. All experiments, unless otherwise indicated, were started 24 h after reaching confluence. At this time-point, basal controls were collected.

Data are shown as mean±standard deviation (SD). Statistical analysis was performed by *t*-test or One-Way ANOVA followed by Holm-Sidak post hoc test when appropriate (SigmaPlot 13.0, Systat Software, Inc., San Jose, CA, USA). A value of p<0.05 was considered as statistically significant.

## 3 Results

### 3.1 High laminar flow protects primary human endothelial cells from the cytotoxic effects of CSEaq

**Fig. 1:**
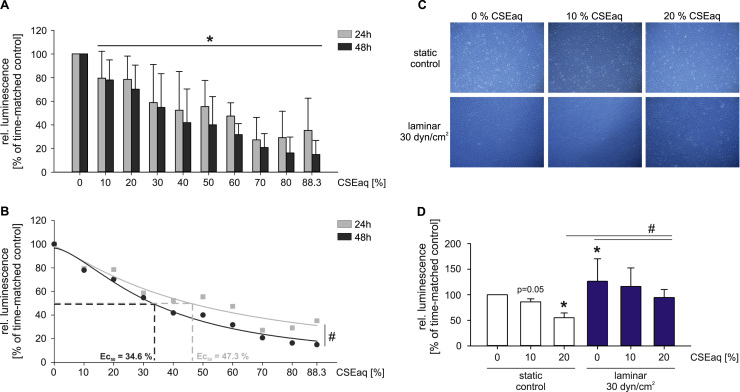
High laminar flow protects primary human endothelial cells from cytotoxic effects of CSEaq. (A) Evaluation of cell viability under static conditions using CellTiter-Glo Luminescent Cell Viability Assay. HUVEC monolayers were stimulated with CSEaq in a concentration range from 10% to 88.3% for 24–48 h. The amount of ATP was determined as an indicator of metabolically active cells and is directly proportional to the number of cells present in culture, n≥6. (B) Calculation of EC_50_ value after exposure to CSEaq. (C) Representative pictures of primary human endothelial cells after 24 h of indicated stimulations are shown, n≥3. (D) Evaluation of HUVEC cell viability under static and high laminar flow conditions and exposure to CSEaq using the ibidi pump system, n≥3. Data are shown as mean (% of time-matched controls) ±SD. *p<0.05 vs. time-matched controls, #p<0.05.

First, we studied putative cytotoxic effects of CSEaq. Primary human endothelial cells (HUVEC) were exposed to increasing dosages of CSEaq (10%–88.3%) for up to 48 h. Treatment with 10% or higher dosages of CSEaq reduced endothelial cell viability in a dose-dependent manner compared to unstimulated controls. The EC_50_ values were calculated using the nonlinear regression analysis of the dose-response curves (47.3% CSEaq after 24 h and 34.6% CSEaq after 48 h). (Fig. 1A, B). Therefore, a maximal CSEaq concentration of 50% was used in all subsequent experiments for 24 h. Application of high laminar flow (30 dyn/cm^2^) caused elongation of the cells in the direction of flow. This was unaffected by CSEaq exposure (Fig. 1C). High laminar flow ameliorated the cytotoxic effects of CSEaq exposure compared to static conditions (Fig. 1D).

### 3.2 CSEaq activates expression of genes involved in endothelial dysfunction and atherosclerosis

**Fig. 2:**
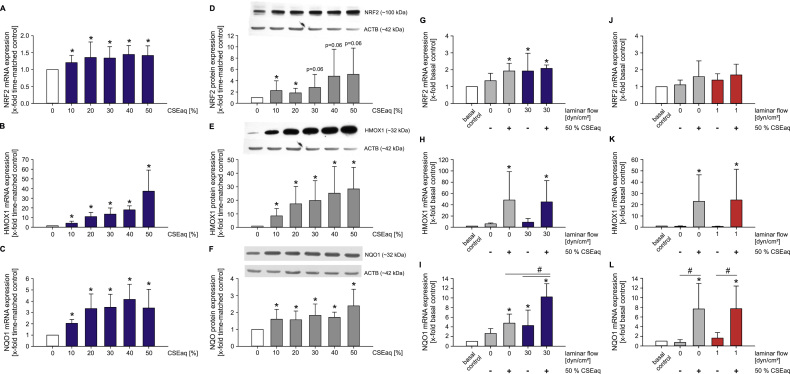
CSEaq leads to activation of NRF2 antioxidant defense system in a dose-dependent manner. Cobblestone cell layers of HUVEC were stimulated with CSEaq under indicated conditions for 24 h. (A–F) mRNA and protein expression of NRF2 (nuclear factor, erythroid 2-like 2); HMOX1 (heme oxygenase 1); NQO1 (NAD(P)H quinone dehydrogenase 1) were determined, n≥7. mRNA expression of (G) NRF2; (H) HMOX1; **(I)** NQO1 after exposure to 50% CSEaq and subjected to laminar flow of 30 dyn/cm^2^, n≥6. (J-L) mRNA expression of NRF2 and its target genes after long-term stimulation with 50% CSEaq and low laminar flow (1 dyn/cm^2^), n≥6. mRNA expression was measured by real-time PCR using POLR2A as reference gene. Protein expression was analyzed by Western Blot using ACTB as loading control. Data are shown as mean (x-fold of time-matched controls (A–F) or basal controls (J-L))±SD. Time-matched controls are normalized to basal controls, where both are shown (G–L). *p<0.05 vs. time-matched controls (A–F) or basal controls (J–L), #p<0.05.

Next, we analyzed the expression of genes involved in the regulation of cellular oxidative stress and the inflammatory state, as markers of endothelial dysfunction and atherosclerosis. Exposure of HUVEC to CSEaq increased the expression of transcription factor NRF2 and its target genes heme oxygenase 1 (HMOX1) and NAD(P)H quinone dehydrogenase 1 (NQO1) on the mRNA and protein level (Fig. 2A–F). While NRF2 and HMOX1 reached the same level of induction by combined stimulation with CSEaq and high laminar flow (30 dyn/cm^2^), induction of NQO1 was potentiated by both stimuli (Fig. 2G–I). Activation of NRF2 and NQO1 by CSEaq seemed to be constitutive, whereas HMOX1 was strongly upregulated in a dose-dependent manner. Stimulation of CSEaq at low laminar flow showed similar results compared to static conditions (Fig. 2J–L).

**Fig. 3:**
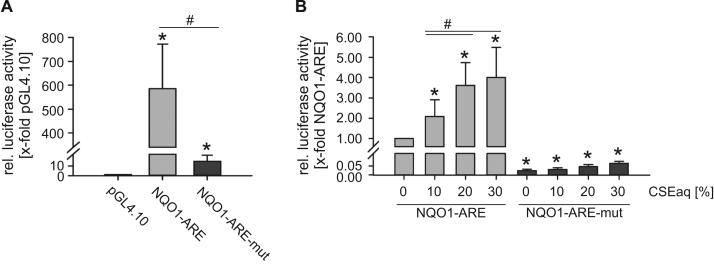
CSEaq activates NQO1 antioxidant response element (ARE) in response to stimulation with CSEaq. Human microvascular endothelial cells (HMEC-1) were transfected with pGL4.10 vector, pGL4.10 containing luciferase reporter gene under control of the cloned human NQO1-ARE or the mutated NQO1-ARE (NQO1-ARE-mut). Cells were transfected for 24 h and activity was measured by Dual-Luciferase Reporter Assay. pGL4.74 was co-transfected for normalization of transfection efficiency. (A) Basal activity of NQO1-ARE and mutated NQO1-ARE-mut compared to the promoterless pGL4.10, n≥4. (B) Determination of luciferase activity after exposure to the indicated amount of CSEaq for 24 h, n≥4. (A) Data are shown as mean (x-fold of pGL4.10 (A) or pGL4.10 NQO1-ARE (B)) ±SD. *p<0.05 vs. pGL4.10 (A) or pGL4.10 NQO1-ARE (B), #p<0.05.

Upon activation, NRF2 binds to antioxidant response elements (ARE) in the 5′ regulatory region of target genes. In order to analyze the underlying molecular mechanism of the regulation by CSEaq, we performed Dual-Luciferase Reporter Assays with the cloned NQO1-ARE and a mutated NQO1-ARE. Both NQO1-ARE clones showed strong basal activity compared to the promoter-less pGL4.10 vector (Fig. 3A). Transfection of human microvascular endothelial cells (HMEC-1) revealed a dose-dependent upregulation of the cloned NQO1-ARE by CSEaq. The mutated NQO1-ARE remains unchanged after stimulation with CSEaq (Fig. 3B).

**Fig. 4:**
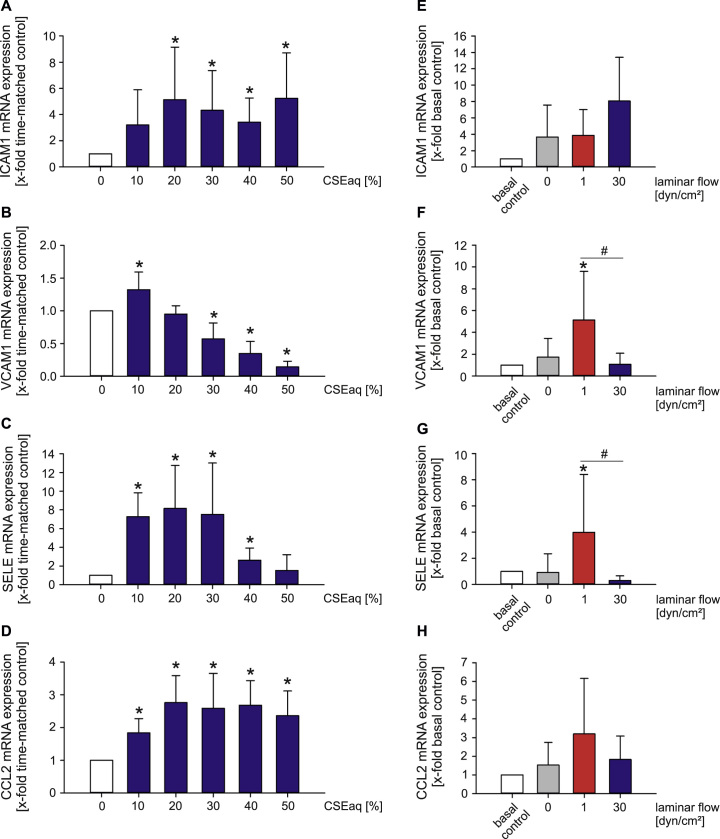
Increased expression of adhesion molecules after stimulation with CSEaq and pro-atherosclerotic low laminar flow. mRNA expression of endothelial (A) ICAM1 (intercellular adhesion molecule-1); (B) VCAM1 (vascular cell adhesion molecule-1); (C) SELE (selectin E); (D) CCL2 (MCP-1/monocyte-chemoattractant protein-1) after dose-dependent stimulation with CSEaq for 24 h under static conditions, n≥6. Flow-dependent regulation of (E) ICAM1; (F) VCAM1; (G) SELE and (H) CCL2 mRNA, n≥7. Primary human endothelial cells (HUVEC) were cultivated until confluence, stimulated with CSEaq in a concentration range from 10% to 50% for up to 24 h or subjected to laminar flow. Subsequently, mRNA expression was measured by real-time PCR. POLR2A was used as reference gene. Data are shown as mean (x-fold of time-matched controls (A–D) or basal controls (E–H)) ±SD. Time-matched controls are normalized to basal controls, where both are shown (E–H). *p<0.05 vs. time-matched controls (A–D) or basal controls (E–H), #p<0.05.

A pro-inflammatory phenotype of endothelial cells is characterized by an increased expression of the adhesion molecules/cytokines ICAM1 (intercellular adhesion molecule-1), CCL2 (MCP-1/monocyte-chemoattractant protein-1), VCAM1 (vascular cell adhesion molecule-1) and SELE (selectin E) [47]. We observed an increased expression of these genes after stimulation with low to medium dosages of CSEaq (Fig. 4A–D). VCAM1 and SELE were regulated in the same manner by atherosclerosis-prone low laminar flow (1 dyn/cm^2^). Both flow conditions did not have a significant impact on ICAM1 and CCL2 expression (Fig. 4E-H).

### 3.3 CSEaq leads to increased adhesion of monocytes to endothelial cells under atherosclerosis-prone low laminar flow

**Fig. 5:**
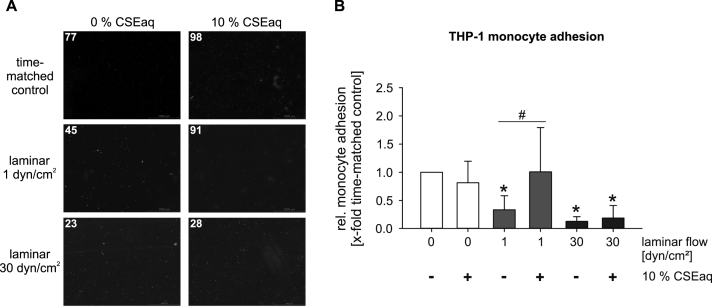
CSEaq leads to increased adhesion of THP-1 monocytes to endothelial cells under atherosclerosis-prone low laminar flow. Monocyte-endothelial cell adhesion assay under different flow conditions using the ibidi pump system. (A) Representative pictures showing monocyte-endothelial interactions. Fluorescently labeled THP-1 monocytes (red, numbers of monocytes are indicated) adhere to a pre-treated confluent endothelial cell layer. Scale bar represents 1000 µm. (B) Evaluation of monocyte-endothelial cell adhesion after exposure to CSEaq in combination with diverse flow conditions. Data are shown as mean (x-fold of time-matched controls)±SD. *p<0.05 vs. time-matched controls, #p<0.05, n ≥6.

Adhesion of monocytes to the endothelium reflects a pro-inflammatory and atherosclerosis-prone phenotype. Despite on the activation of NRF2 antioxidative defense system by CSEaq, stimulation with 10% CSEaq induced an inflammatory phenotype in endothelial cells. This was shown functionally by increased monocyte adhesion to endothelial cells under low laminar flow conditions. In contrast, high laminar flow mediated atheroprotective effects on monocyte adhesion. These protective effects of high laminar flow were able to inhibit pro-inflammatory effects of stimulation with CSEaq. Similar data were obtained using THP-1 monocytes (Fig. 5) and primary human monocytes (see Online data supplement). Representative movies are shown in the online data supplement.

Supplementary material related to this article can be found online at doi:10.1016/j.redox.2017.04.008.

The following is the Supplementary material related to this article Movie 1, Movie 2, Movie 3,.

Movie 1 Representative movie showing monocyte-endothelial adhesion after stimulation of HUVEC with high laminar flow for 72 h. THP-1 monocytes were subjected to pre-stimulated endothelial cells with laminar flow of 1 dyn/cm^2^ for 2 min real-time, n=3.

Movie 2 Representative movie showing monocyte-endothelial adhesion after stimulation of HUVEC with high laminar flow for 24 h reaching a confluent cell layer and subsequent application of laminar flow of 1 dyn/cm^2^ for 48 h. THP-1 monocytes were applied to pre-stimulated endothelial cells at laminar flow of 1 dyn/cm^2^ for 2 min real-time, n =3.

Movie 3 Representative movie showing monocyte-endothelial adhesion after stimulation of HUVEC with high laminar flow for 24 h reaching a confluent cell layer and subsequent application of laminar flow of 1 dyn/cm^2^ for 48 h including an additional exposure to 10% CSEaq for the last 24 h. THP-1 monocytes were subjected to pre-stimulated endothelial cells at laminar flow of 1 dyn/cm^2^ for 2 min real-time, n=3.

### 3.4 Impaired wound healing capability of endothelial cells in response to CSEaq

A wound in the endothelial cell layer is considered as an initial stimulus for the development of atherosclerotic plaques (response-to-injury model). Wound healing of HUVEC monolayers were analyzed in response to CSEaq, low and high laminar flow conditions using a cone-and-plate viscometer. Prior to stimulation with flow, HUVEC showed typical cobblestone morphology with a defined wound area. For determination, areas under the curves were evaluated.

**Fig. 6:**
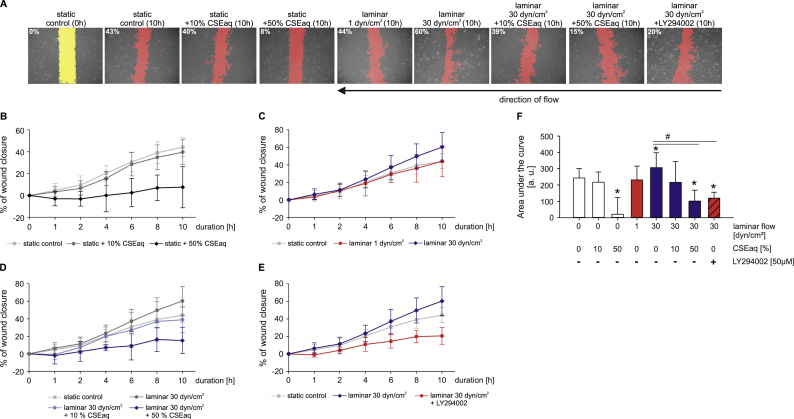
Protective effects of high laminar flow on wound healing are inhibited by CSEaq and PI3K/AKT/eNOS pathway inhibitor. (A) Representative pictures of wound healing after 10 h including degree of wound healing in % are shown. Determination of increased wound closure of primary human endothelial cells in response to (B) increasing CSEaq dosages; (C) diverse flow conditions; (D) high laminar flow of 30 dyn/cm^2^ in combination with CSEaq; (E) high laminar flow of 30 dyn/cm^2^ in combination with LY294002 using a cone-and-plate viscometer. Direction of flow was perpendicular to the wound in the cell layer. Data are shown as mean (% of wound closure) ±SD. n≥6. (F) Evaluation of area under the curve data of wound healing capability under indicated stimulation. Data are shown as mean (AUC) ±SD. *p<0.05 vs. static time-matched controls, #p<0.05.

Stimulation with high laminar flow led to significantly accelerated wound closure. Stimulation with low laminar flow showed no effect on wound closure. Remarkably, additional CSEaq treatment inhibited positive effects of high laminar flow on wound healing in a dose-dependent manner. The relative area of endothelial wound closure under static and low laminar flow was comparable to the extent of wound closure following stimulation with CSEaq. Inhibition of PI3K/AKT/eNOS pathway by LY294002 inhibitor resulted in comparable adverse effects on wound healing as CSEaq stimulation after 10 h of treatment (Fig. 6).

### 3.5 Impact of CSEaq and laminar flow on PI3K/AKT/eNOS signaling in endothelial cells

**Fig. 7:**
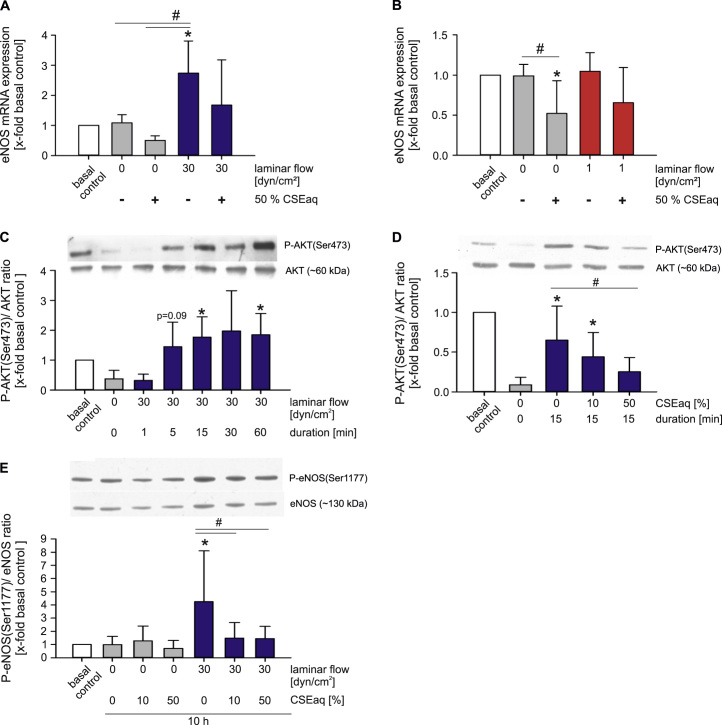
Protective activation of PI3K/AKT/eNOS signaling pathway by high laminar flow is inhibited by CSEaq. (A–B) mRNA expression of eNOS (endothelial nitric oxide synthase) in HUVEC exposed to 50% CSEaq after subjected to high or low laminar flow conditions for 7 h, n≥6. (C) Phosphorylation status of AKT pathway in primary human endothelial cells after short-time stimulation with high laminar flow. Cells were resting in basal media without supplements for 60 min prior stimulation, n≥6. (D) Re-phosphorylation of AKT after resting in response to increasing dosages of CSEaq, n≥5. (E) eNOS phosphorylation status after 10 h of CSEaq treatment under static conditions and high laminar flow, n≥6. Data are shown as mean (*x*-fold basal controls) ±SD. Time-matched controls are normalized to basal controls as well. *p<0.05 vs. basal controls, #p<0.05.

Phosphorylation of PI3K/AKT/eNOS pathway in endothelial cells in response to laminar flow is a protective event, leading to increased eNOS activation. We could show a significantly increased eNOS expression after stimulation with high laminar flow for 24 h, compared to static controls and low laminar flow (see Online data supplement). In addition, HUVEC subjected to high laminar flow showed a rapid activation of the PI3K/AKT/eNOS pathway via phosphorylation of AKT (Ser473) (Fig. 7C). This resulted in a strongly increased NO release under high laminar flow conditions after 24 h. Interestingly, long term application of low laminar flow slightly increased NO release as well (see Online data supplement). CSEaq reduced mRNA expression of eNOS (Fig. 7A–B) and diminished the capability of re-phosphorylation of AKT (Ser473) in a dose-dependent manner after a resting period of 60 min (Fig. 7D). Stimulation of HUVEC with CSEaq under high laminar flow resulted in reduced eNOS phosphorylation at Ser1177 compared to the same flow without CSEaq (Fig. 7E). The PI3K inhibitor LY294002 served as control and completely abrogated AKT phosphorylation at Ser473 (see Online data supplement).

## 4 Discussion

Endothelial dysfunction and atherosclerosis are caused by a wide range of risk factors. One of the most important behavioral risk factor is tobacco consumption [8]. The molecular mechanisms that underpin how cigarette smoke affects endothelial function are part of intense research. The data generated within this study incrementally advances our understanding of endothelial dysfunction in response to cigarette smoke. An interesting model to study smoking-related cellular changes is the application of CSEaq, which captures both particulate and vapour phase components of cigarette smoke, to cellular systems *in vitro* [48]. The hemodynamic environment of endothelial cells *in vivo* plays also a major role in the maintenance of a physiological endothelial phenotype. To investigate the impact of laminar flow on the biological response of endothelial cells to cigarette smoking, we systematically studied the impact of CSEaq in combination with atheroprotective high laminar (30 dyn/cm^2^) or pro-atherosclerotic low laminar (1 dyn/cm^2^) flow.

Our *in vitro* model revealed a dose-dependent reduction in endothelial cell viability in response to CSEaq from 3R4F reference cigarettes at concentrations higher than 10%. Similar dosages were used in recent studies investigating the time- and dose-dependent induction of endothelial autophagic cell death or apoptosis by CSEaq generated from filtered [49] or unfiltered cigarettes [50]. We were able to demonstrate beneficial effects of high laminar flow on endothelial viability which counteracts the cytotoxic potential of CSEaq treatment and delay the progression of cell death. The shown elongation of endothelial cells in the direction of flow reflects the native phenotype of endothelial cells in high flow areas of the vasculature [51].

We also analyzed changes of different biomarkers such as the NRF2 antioxidant defense system, adhesion molecules and cytokines, known to be representative for an anti-oxidative and pro-inflammatory expression profile of endothelial cells. By studying the NRF2 system, we observed an increased expression of transcription factor NRF2 and its target genes HMOX1 and NQO1 mediated by CSEaq treatment which is strongly supported by previous research showing induction of NRF2, HMOX1 and NQO1 by CSEaq in several cellular systems [52], [53], [54], [55], [56]. As none of these previous studies analyzed the impact of laminar flow on these responses, we analyzed the expression of the NRF2 system under flow conditions in combination with CSEaq. NRF2 is known as a mechanosensitive transcription factor in endothelial cells [57] and there is growing evidence that supports NRF2-mediated atheroprotective and anti-oxidative effects in response to laminar flow [58], [59], [60]. Our own previous studies showed a translocation of NRF2 into the nucleus in response to atheroprotective high laminar flow [37] which is underpinned by an increased NRF2 translocation shown in the endothelium in atherosclerosis-resistant regions of the mouse aorta [61]. In the present study, application of high laminar flow was leading to an activation of the NRF2 pathway and an increased expression of NRF2 target genes in human endothelial cells, whereas low laminar flow induced no changes in expression. We identified no additional impact on the CSEaq-dependent induction of NRF2 expression and its target genes under laminar flow conditions. However, the increased NRF2 expression might mediate anti-oxidative and vasoprotective effects in an attempt to compensate the deleterious effects of CSEaq on endothelial cells. On the contrary, Warabi et al. showed a regulation of NRF2-regulating genes as HMOX1 and NQO1 under low laminar flow suggesting that the most critical determinant for the induction of an anti-atherogenic and cytoprotective expression profile is the direction of flow rather than its strength [62], [63]. Taking both under consideration we may argue that the effect of laminar flow in terms of quality and quantity on endothelial cells is still a subject for further debate.

In order to elucidate the underlying molecular mechanisms of NRF2 signaling in more detail, we studied the impact of CSEaq on the NQO1 promoter activity. Stimulation with CSEaq induced activity of the NQO1-NRF2 binding antioxidant response element (ARE) in human endothelial cells. Mutation of the NQO1-ARE blunted this response to CSEaq. Our data strongly support an important role of this ARE in the induction of NQO1 gene expression in human endothelial cells which is in agreement with previous studies using pro-inflammatory and pro-atherogenic oxidized phospholipids [64], the flavonol quercetin [65] and an extract of Ginkgo biloba [66].

A crucial step in vascular inflammation and atherosclerosis is the adhesion of monocytes/macrophages to the endothelium [30]. We found an induction of adhesion molecules ICAM1, VCAM1 and SELE by CSEaq in human endothelial cells similar to previous studies [67], [68]. Serum of smokers increased the expression of ICAM1 and the monocytes adhesion to endothelial cells under static conditions [69]. On the other hand, the expression of adhesion molecules in endothelial cells also depends on the type, duration and magnitude of hemodynamic forces. In the present study, high laminar flow induced atheroprotective effects on VCAM1 and SELE expression, whereas pro-atherogenic low laminar flow increased gene expression, similar to the effects of low doses of CSEaq. These findings are strongly supported by other research showing dose-dependent downregulation of VCAM1 after 6 h of high flow via an AP-1 site in its promoter in murine endothelial cells [70] or upregulated ICAM1, VCAM1 and SELE expression under pro-atherosclerotic low flow in human retinal microvascular endothelial cells [71]. The shown induction of CCL2 expression in endothelial cells after CSEaq stimulation suggests a pro-inflammatory effect of CSEaq promoting the activation of the endothelium and further the adhesion of monocytes/macrophages to endothelial cells. Furie et al. published similar results with elevated adhesion molecule SELE and monocyte chemoattractant protein-1 (MCP-1/CCL2) using extracts of smokeless tobacco [72].

To advance the physiological understanding of endothelial phenotypic conversion to a state that facilitates adherence of leukocytes, we investigated monocyte adhesion to endothelial cells in response to CSEaq under high and low laminar flow. Whereas treatment with 10% CSEaq increased monocyte adhesion under low laminar flow, high laminar flow reduced monocyte adhesion per se and prevented increased adhesion under stimulation with CSEaq as well. Similar data were obtained with THP-1 cells and primary human monocytes, suggesting a different effect using a monocytic cell line or primary human cells. Therefore, we suggest that the deleterious effects of CSEaq treatment on monocyte adhesion can be inhibited by high laminar flow to a certain degree. Previous studies described an increased monocyte adhesion to endothelial cells in response to CSEaq under static conditions [67]. A positive correlation has been found between monocyte adhesion to endothelial cells and the smoking status of patients with atherosclerosis [73], [74]. This is in agreement with studies showing increased monocyte adhesion in response to smoking and improved endothelial dysfunction by oral supplementation with vitamin C in human smokers [75], [76].

A wound in the endothelial cell layer is considered as an initial stimulus for the development of atherosclerotic lesions [15]. In a wound healing assay, we analyzed the migration behavior of cells under various treatments. Application of high laminar flow enhanced wound closure of endothelial cell layer compared to static and low laminar flow conditions. Physiological levels of laminar flow enhanced endothelial repair in a dose-dependent manner likewise [77]. Additional treatment with CSEaq prevented these protective effects of high laminar flow in a dose-dependent manner. This is in agreement with the impaired wound healing observed in smokers [78] and inhibitory effects of CSEaq on wound repair of porcine aortic endothelial layer [79].

Increased phosphorylation and expression of the PI3K/AKT/eNOS pathway plays a substantial role in mediating the protective effects of high laminar flow in endothelial cells [26], [27]. These protective effects of high laminar flow on wound healing might involve the flow-dependent release of NO [80] and it is known that phosphorylation of eNOS and the survival kinase AKT are key mechanisms in the protection of endothelial cells by high laminar flow [27]. There is evidence that reduced NO and excessive ROS formation due to impairment of eNOS phosphorylation are a major factors contributing to a declined wound healing capability [81]. In our study, PI3K inhibition caused comparable adverse effects on wound healing as CSEaq stimulation. Therefore, we analyzed the role of the PI3K/AKT/eNOS pathway in CSEaq-impaired wound healing in combination with different types of flow in more detail. In agreement with previous studies, we found an increased eNOS expression, phosphorylation at Ser1117 and NO release in response to high laminar flow [82], [83], [84]. Furthermore, high laminar flow rapidly activated AKT phosphorylation at Ser473, whereas CSEaq inhibited the vasoprotective eNOS and AKT phosphorylation in response to high laminar flow. A similar response was observed after PI3K inhibition by LY294002. Therefore, inhibition of the PI3K/AKT/eNOS pathway might be a key mechanism in the CSEaq-mediated abrogation of vasoprotective effects of high laminar flow on endothelial cells.

## 5 Conclusions

This study suggests that stimulation of endothelial cells with cigarette smoke leads to an activation of key parameters, known to be involved in the development of endothelial dysfunction and atherogenesis. Likewise, a delayed response to the harmful effects of cigarette smoking under high laminar flow has to be expected. Even though, atheroprotective high laminar flow did not completely abrogate the detrimental effects of stimulation with CSEaq, the effects were clearly delayed. The revealed data strongly suggest the PI3K/AKT/eNOS pathway as a key mechanism involved in the CSEaq-induced inhibition of atheroprotective effects of high laminar flow and needs further investigation in future studies.

## Compliance with ethical standards

This study is approved by the ethical review board of the Medical Faculty Carl Gustav Carus of the TU Dresden (EK124082003).

## Conflict of interest

On behalf of all authors, the corresponding author states that there is no conflict of interest.

## Figures and Tables

**Table 1:** Primers used for analysis of human gene expression by real-time PCR.

**Target gene**	**Primer**	**Sequence (5´→3´)**
**POLR2A**	Sense	ACCTGCGGTCCACGTTGTGT
	Antisense	CCACCATTTCCCCGGGATGCG
**eNOS (NOS3)**	Sense	GAACCTGTGTGACCCTCACC
	Antisense	TGGCTAGCTGGTAACTGTGC
**NRF2 (NFE2L2)**	Sense	CCCAATTCAGCCAGCCCAGC
	Antisense	AACGGGAATGTCTGCGCCAA
**HMOX1**	Sense	CGGATGGAGCGTCCGCAACC
	Antisense	TCACCAGCTTGAAGCCGTCTCG
**NQO1**	Sense	CCCCGGACTGCACCAGAGC
	Antisense	CTGCAGCAGCCTCCTTCATGGC
**ICAM1**	Sense	ACCATGGAGCCAATTTCTCG
	Antisense	GCGCCGGAAAGCTGTAGATG
**CCL2 (MCP-1)**	Sense	GCTCAGCCAGATGCAATCA
	Antisense	TTTGCTTGTCCAGGTGGTC
**VCAM1**	Sense	TGTGCCCACAGTAAGGCAGGC
	Antisense	AGCTGGTAGACCCTCGCTGGA
**SELE**	Sense	AGCCCAGAGCCTTCAGTGTA
	Antisense	CTCCAATAGGGGAATGAGCA

Abbreviations: POLR2A: RNA polymerase II subunit A; eNOS/NOS3: endothelial NO synthase; NRF2/NFE2L2: nuclear factor erythroid 2-related factor 2; HMOX1: heme oxygenase (decycling) 1; NQO1: NAD(P)H quinone dehydrogenase 1; ICAM1: intercellular adhesion molecule-1; CCL2: chemokine (C-C motif) ligand 2/MCP-1: monocyte-chemoattractant protein-1; VCAM1: vascular cell adhesion molecule-1; SELE: selectin E.
