# Supplementary material for: Cigarette smoke extract counteracts atheroprotective effects of high laminar flow on endothelial function
Source: Redox Biol. 2017 Apr 7;12:776–86. doi: 10.1016/j.redox.2017.04.008 (PMC5397582; doi:10.1016/j.redox.2017.04.008)
Supplement: Supplementary file 4 — Supplementary material [file mmc1.docx]

**Online Data Supplement**

**Cigarette smoke extract counteracts atheroprotective effects of high laminar flow on endothelial function**

Sindy Giebe^1^, Natalia Cockcroft^2^, Katherine Hewitt^2^, Melanie Brux^1^, Anja Hofmann^1^, Henning Morawietz^1*^, Coy Brunssen^1*^

*^1^Division of Vascular Endothelium and Microcirculation, Department of Medicine III, University Hospital Carl Gustav Carus Dresden, Technische Universität Dresden, Dresden, Germany*

*^2^Research & Development, British American Tobacco, Southampton, United Kingdom*

*Corresponding authors:

Coy Brunssen, PhD; Division of Vascular Endothelium and Microcirculation, Department of Medicine III, University of Technology Dresden, Fetscherstr. 74, D-01307 Dresden, Germany.

Phone.: +49-351-458-6677; fax: +49-351-458-6354

E-mail: Coy.Brunssen@uniklinikum-dresden.de

Henning Morawietz, PhD; Division of Vascular Endothelium and Microcirculation, Department of Medicine III, University of Technology Dresden, Fetscherstr. 74, D-01307 Dresden, Germany.

Phone.: +49-351-458-6625; fax: +49-351-458-6354

E-mail: Henning.Morawietz@uniklinikum-dresden.de

**
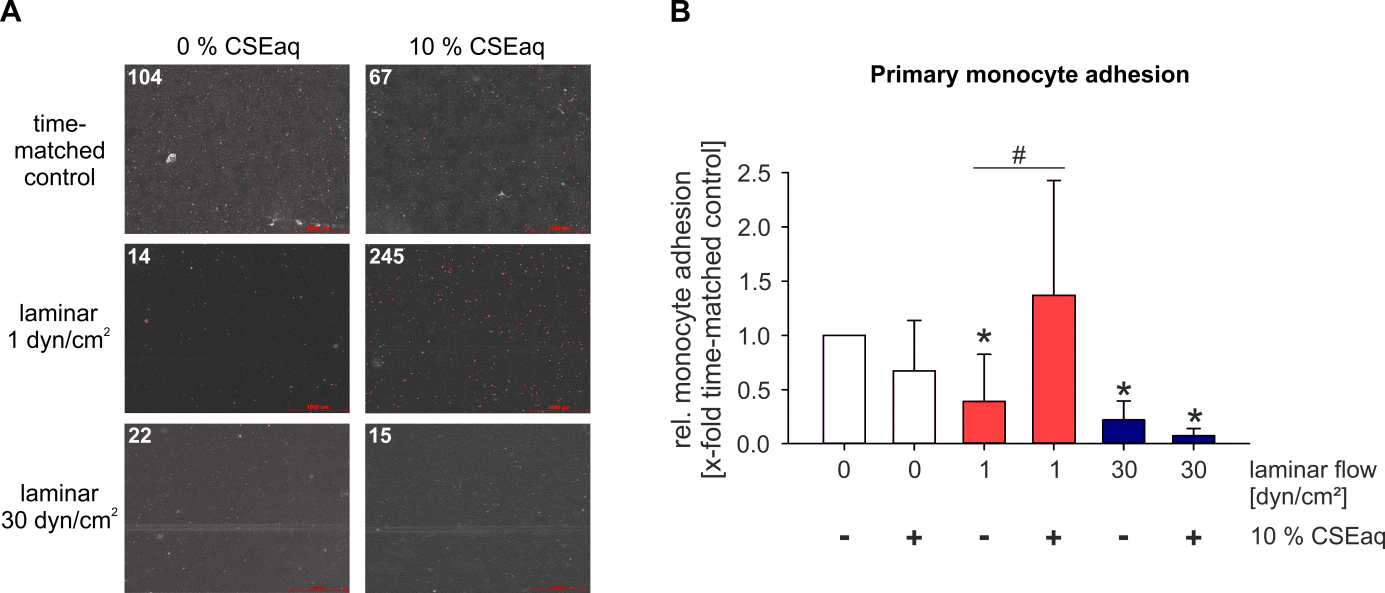
**

**Fig. S1.** CSEaq leads to increased adhesion of primary monocytes to endothelial cells under atherosclerosis-prone low laminar flow. Monocyte-endothelial cell adhesion assay under different flow conditions using ibidi pump system. (A) Representative pictures showing monocyte-endothelial interactions. Fluorescently labelled primary monocytes (red, numbers of monocytes are indicated) adhere to a pre-treated confluent endothelial cell layer. Scale bar represents 1000 µm. (B) Evaluation of monocyte-endothelial cell adhesion after exposure to CSEaq in combination with diverse flow conditions. Data are shown as mean (x-fold of time-matched control) ± SD. *p < 0.05 vs. time-matched control, #p < 0.05, n ≥ 6.

**
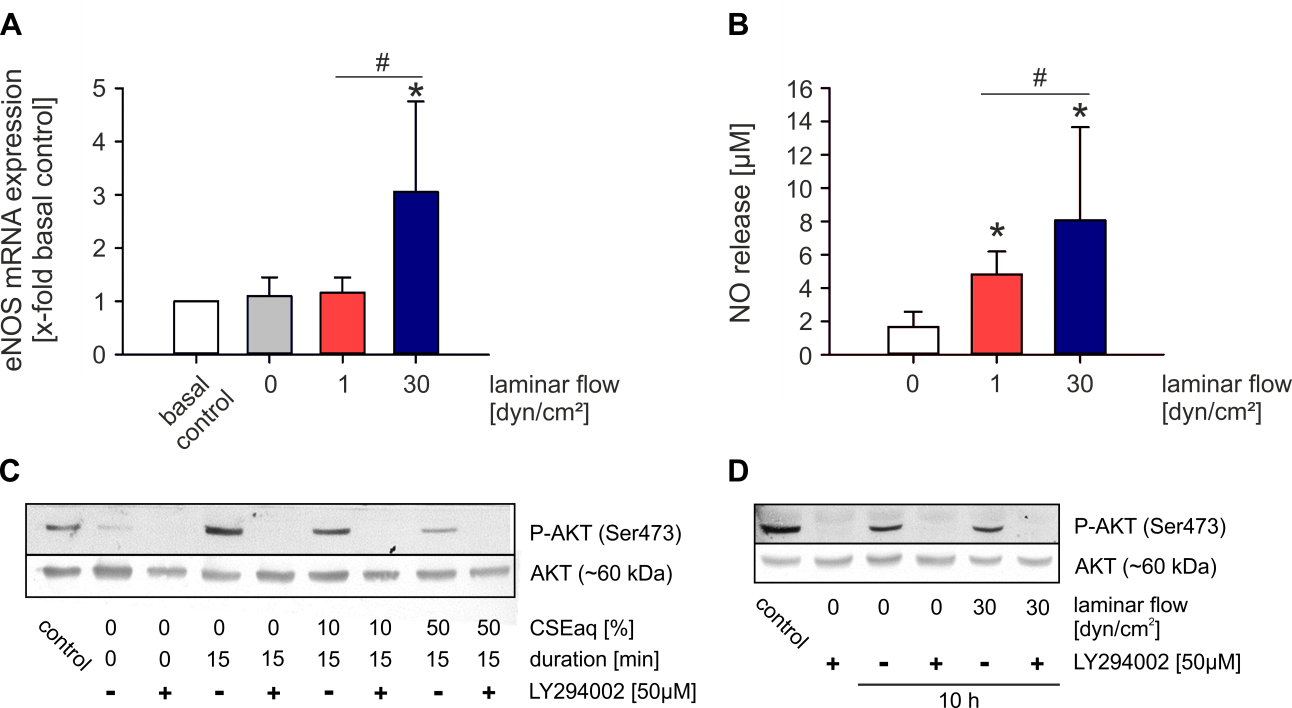
**

**Fig. S2.** Protective activation of PI3K/AKT/eNOS signaling pathway by high laminar flow is inhibited by CSEaq. (A) mRNA expression of eNOS (endothelial nitric oxide synthase) in HUVEC after exposure to different flow conditions for 24 h, n ≥ 6. (B) Regulation of endothelial nitric oxide (NO) release in response to flow. NO release was determined by Griess assay, n ≥ 9. (C) Re-phosphorylation of AKT in response to increasing dosages of CSEaq. Activation of PI3K/AKT/eNOS signaling pathway after resting is completely inhibited by stimulation with PI3K inhibitor LY294002, n ≥ 3. (D) AKT phosphorylation status in HUVEC after stimulation for 10 h with PI3K inhibitor LY294002 under static conditions and high laminar flow, n ≥ 4. (A) Data are shown as mean (x-fold of basal control (A)) ± SD. Time-matched controls are normalized to basal controls (A+B). *p < 0.05 vs. basal control (A) or time-matched control (B), #p < 0.05.

**Fig. S3.** Representative movie showing monocyte-endothelial adhesion after stimulation of HUVEC with high laminar flow for 72 h. THP-1 monocytes were subjected to pre-stimulated endothelial cells with laminar flow of 1 dyn/cm^2^ for 2 min real-time, n = 3.

**Fig. S4.** Representative movie showing monocyte-endothelial adhesion after stimulation of HUVEC with high laminar flow for 24 h reaching a confluent cell layer and subsequent application of laminar flow of 1 dyn/cm^2^ for 48 h. THP-1 monocytes were applied to pre-stimulated endothelial cells at laminar flow of 1 dyn/cm^2^ for 2 min real-time, n = 3.

**Fig. S5.** Representative movie showing monocyte-endothelial adhesion after stimulation of HUVEC with high laminar flow for 24 h reaching a confluent cell layer and subsequent application of laminar flow of 1 dyn/cm^2^ for 48 h including an additional exposure to 10 % CSEaq for the last 24 h. THP-1 monocytes were subjected to pre-stimulated endothelial cells at laminar flow of 1 dyn/cm^2^ for 2 min real-time, n = 3.
